# Preserving Human Judgment in AI-Enabled Education: The SAM Model for AI Readiness, Assessment, and Human-in-the-Loop Decision Making

**DOI:** 10.3390/bs16101938

**Published:** 2026-10-18

**Authors:** Holger Horz, Pauline Czora, Julia Mendzheritskaya, Pauline Naumann, Maria Zirenko

**Affiliations:** Department of Educational Psychology, Institute of Psychology, Goethe University Frankfurt, Theodor-W.-Adorno-Platz 6, 60629 Frankfurt am Main, Germany; czora@psych.uni-frankfurt.de (P.C.); pa.naumann@psych.uni-frankfurt.de (P.N.); zirenko@psych.uni-frankfurt.de (M.Z.)

**Keywords:** artificial intelligence in education (AIEd), learning analytics, construct validity, human-in-the-loop, AI-based assessment, educator AI readiness, self-regulation, ambiguity tolerance, methodological competence, algorithmic fairness

## Abstract

Generative artificial intelligence (AI) shifts educational focus from access to information toward judging machine-generated claims and regulating the division of epistemic work. This conceptual article proposes the SAM model—self-regulation (S), ambiguity tolerance (A), and methodological competence (M)—as a bounded person-level resource model within broader AI readiness, with educators’ AI readiness as a central application. S denotes regulatory capacity, A a disposition-like willingness to tolerate unresolved epistemic ambiguity, and M the capacity to evaluate claims, differentiated into cross-domain M1 and domain-specific M2. SAM’s distinctive claim is configurational: M should predict independently assessed judgment more strongly at higher S and, under high ambiguity, when A is higher. These relations, the M1/M2 distinction, limited compensability, and incremental prediction beyond shared and neighboring accounts remain hypothetical. The model separates resources from enacted processes, contextual affordances, and outcomes and requires independent performance criteria. For AI-based assessment and learning analytics, educators’ interpretations of AI-derived evidence and construct inferences from behavioral traces require validated mappings addressing alternative explanations, comparability, fairness, and use. SAM thus offers a research agenda predicting when AI support preserves human judgment rather than merely improving supported performance.

## 1. Introduction: From AI Literacy to Preserving Human Judgment

Generative artificial intelligence (AI) changes not only access to information but also the distribution of epistemic work. Formulations, hypotheses, explanations, and lines of reasoning can be generated with little effort. The challenge is no longer information scarcity. What becomes scarce are the capacities to set one’s own goals, calibrate support, tolerate uncertainty, and evaluate machine-generated claims. The educational–psychological question is therefore which person-level resources enable people to preserve warranted judgment in coupled human–AI cognition, and how their contribution can be distinguished from merely supported performance. This problem becomes particularly visible in AI-based assessment and learning analytics, where adaptive assessment systems, behavioral traces, and automated indicators increasingly inform educational judgments.

AI literacy and readiness frameworks already address technical, evaluative, social, and ethical demands (Chiu, 2025; Long & Magerko, 2020; Wang et al., 2023), while critical thinking in AI use offers a directly relevant account of evaluation (Lau et al., 2026). SAM asks whether self-regulation (S), ambiguity tolerance (A), and methodological competence (M, differentiated into cross-domain M1 and domain-specific M2) form a configuration that explains professionals’ interpretation of AI-derived evidence and predicts independently assessed judgment. Its contribution is the distinction between regulatory capacity, willingness to tolerate unresolved ambiguity, and methodological application capacity, together with the prediction that the contribution of M to independently assessed judgment varies with S and, under ambiguity, depends on A. We view the three constructs as operating at the same conceptual level rather than as hierarchically organized; their relations remain to be tested empirically.

### 1.1. The VUCA World and the Moving-Target Problem

**Figure 1:**
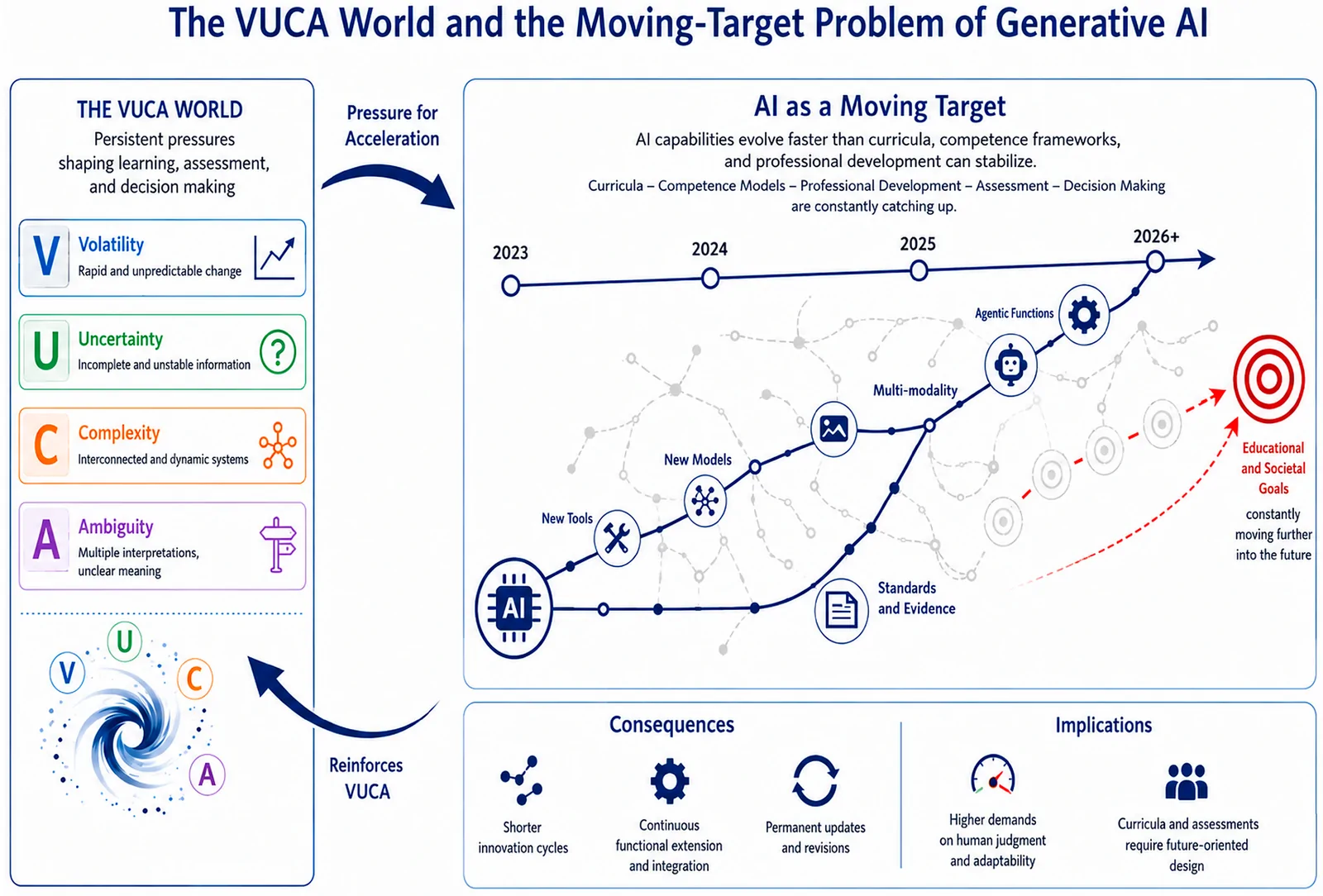
The VUCA World and the Moving-Target Problem of Generative AI. Accelerated AI development intensifies volatility, uncertainty, complexity, and ambiguity and creates shorter innovation cycles, continuing functional extension, and repeated revision; educational responses therefore require human judgment, adaptability, and future-oriented competence. This is a schematic illustration; the curves and dates do not represent measured trajectories or forecasts.

VUCA denotes environments marked by volatility, uncertainty, complexity, and ambiguity, in which rapid change, incomplete information, interdependence, and multiple plausible interpretations constrain conventional planning and decision making (Bennett & Lemoine, 2014). Generative AI compounds these pressures: models, multimodal functions, and agentic systems change faster than curricula, competence models, and professional-development programs can stabilize. These VUCA conditions arise not only at the level of societal transformation but directly within learning, assessment, and decision-making situations (Horz & Czora, 2026). Figure 1 summarizes how accelerated AI development shortens the usable lifespan of tool-specific curricula and competence catalogs, requiring reference points that remain meaningful as interfaces, functions, and model generations change.

This moving-target problem is not an argument against AI literacy or specific AI-related competencies; European and international frameworks appropriately identify institutional requirements (European Parliament & Council of the European Union, 2024; Kultusministerkonferenz, 2024; Miao & Cukurova, 2024; OECD & European Commission, 2026). It is, however, an argument against curricula tied primarily to tool names, prompting routines, or current interfaces. A more temporally robust reference point is AI readiness (e.g., Wang et al., 2023), understood as the adaptive willingness and capability to apply and further develop AI-related knowledge and competencies under changing conditions.

Within this broader readiness perspective, SAM specifies three core educational–psychological resources. The guiding question shifts from “Which tool must be mastered today?” to “Which human resources will still safeguard judgment tomorrow?”.

### 1.2. Epistemological Positioning and Model Architecture

SAM is a parsimonious, functionally differentiated psychological resource model, not a hierarchical trait model or additive competence catalog. Different construct types serve distinct functions in epistemic control. We use epistemic control to denote the extent to which judgment-defining epistemic functions (setting goals, deciding what to delegate, evaluating claims and making accountable final judgments) are actually exercised by the human in human–AI interaction. Epistemic control is therefore not a person-level resource but a property of the interaction. Cummins’s (1975) functional-analysis logic analyzes complex capacities through organized subfunctions enabling overall performance; SAM accordingly selects components for explanatory function rather than descriptive completeness. Selection followed three functional criteria: a distinct judgment-defining function in epistemic control, assessability at the person level independently of a single episode, and plausible nonredundancy with the other resources. Accounts that explain conditions of exercise, interaction architecture, or assessment inference were assigned contextual, architectural, or inferential roles instead (Section 4).

The architecture distinguishes distinct resource functions, potentially interdependent contributions, and limited compensation for a weak resource. Interactions and the limited extent to which one component can compensate for another require separate tests (Section 3.5.1); neither implies a common higher-order latent capacity.

### 1.3. Theoretical Gap and Aims of the Paper

This paper specifies the resource configuration and its relation to regulatory control in human–AI interaction to preserve human judgment, defines how performance could warrant resource inferences, and derives predictions against independently justified outcomes and competing models. Separating resources, enactment, and success connects SAM to AI-based assessment: observed activity provides evidence about a resource only through a justified inference, and resource assessment and success criteria remain distinct.

SAM is a bounded psychological resource model nested in broader AI readiness: it specifies three person-level resources and their hypothesized interplay rather than offering a complete account of literacy, readiness, or responsible AI use. Domain knowledge, foundational skills, ethical standards, relationships, and institutional opportunities remain relevant at different analytical levels. Human–AI interaction is the umbrella term; collaboration denotes joint task performance, hybrid regulation the distribution of regulatory functions, and sociotechnical coupling the architecture of coupled human–system performance. Functional collaboration implies neither symmetrical social nor intentional agency.

This functionally guided theoretical synthesis uses recent research to define resources, specify boundaries, and inform measurement claims. Section 2, Section 3 and Section 4 address the problem, resources, and boundaries; Section 5 and Section 6 measurement and tests; Section 7 and Section 8 assessment, professional, organizational, and equity implications.

## 2. Human–AI Interaction as a Problem of Epistemic Regulation

### 2.1. Distributed Cognition, Agency, and Epistemic Authorship

Research on distributed cognition and joint cognitive systems shows that performance can be distributed across people, artifacts, and social arrangements (Hollan et al., 2000; Hollnagel & Woods, 2005; Hutchins, 1995). Generative AI extends this configuration by generating proposals, hypotheses, priorities, and reasoning patterns. Performance increasingly requires coordination of internal knowledge, external representations, and calibrated trust (Lee & See, 2004), changing the division of knowledge production and evaluation.

Education requires person, task, system, and developmental-level fit, not system capability alone; person–program research supports this general view (Horz, 2004; Horz & Ulrich, 2022). External collaboration scripts and learners’ existing argumentation knowledge can relate differently to learning outcomes (Kollar et al., 2007). In the context of AI, this raises the question of which epistemic functions are offloaded, retained, or reorganized by external structures and how that distribution is controlled. SAM describes Person × System × Task × Developmental Level without prescribing a statistical four-way interaction. This interactionist assumption accords with Lewin’s (1936) B = f(P, E): behavior depends on the person and psychologically relevant environment.

Bandura (2001) characterizes human agency through four interrelated functions: intentionality, forethought, self-reactive regulation of one’s actions, and reflective self-observation. Agency is therefore more than freedom of choice; it is the capacity to initiate goal-directed action, anticipate consequences, align one’s behavior with standards, and reflect on one’s decisions. In AI environments, epistemic agency can be derived as a specific form of agency: setting one’s own goals, determining the type and extent of delegation to AI, evaluating machine suggestions, revising decisions in light of new evidence, and assuming responsibility for the final reasoned judgment. Parasuraman and Riley (1997) distinguish use, misuse, disuse, and abuse of automation; whether automation relieves cognitive demands or weakens human control depends on the balance among overreliance, under-reliance, and inappropriate task allocation.

Descriptively, regulatory locus is an empirical question: Who sets goals, monitors, adapts strategies, and decides task completion in a human–AI configuration? Normatively, ultimate epistemic responsibility should remain with humans. This commitment is an educational–psychological quality criterion, not an empirical finding; its conditions and consequences require testing.

Externally supported performance is not automatically internalized (Vygotsky, 1978). We reserve scaffold for support that prepares or extends independent performance and permits responsibility to return as competence develops. Pea (2004) cautions against calling every technological aid scaffolding: improved short-term AI performance is insufficient, and persistent substitution is not scaffolding when the learner no longer develops or cannot resume the target operation independently.

SAM relates its resource hypotheses to epistemic control without equating a correct AI-assisted product with human authorship. Intellectual sovereignty refers to the capacity to use cognitive support without relinquishing epistemic authorship and reasoned judgment to the system (Wissenschaftsrat, 2026).

### 2.2. Cognitive Offloading, Appropriate Reliance, and Human-in-the-Loop

The offloading literature treats the use of external tools as potentially productive cognitive division of labor, not loss by definition (Risko & Gilbert, 2016). Its value depends on the task, learning phase, and target competence. Building on calibrated trust (Lee & See, 2004), appropriate reliance in SAM concerns whether accepting, rejecting, verifying, or delegating support fits those requirements. Overreliance denotes reliance beyond what the available evidence and task requirements warrant. Frequency of use, expressed trust, and an incorrect answer are not interchangeable indicators of it; the person’s information, choices, and opportunities to check must be considered.

Empirical findings distinguish perceived credibility from actual uptake. Dahlkemper et al. (2023) found that an inadequate ChatGPT (ChatGPT 3.5) response to a difficult physics item was rated relatively close to an expert response, although the expert response still received higher scientific-accuracy ratings while linguistic-quality ratings did not differ significantly; fluency itself was not independently manipulated. Urban et al. (2025) found that ChatGPT assistance increased correct-information uptake without significantly changing incorrect-information uptake, and that higher metacognitive bias was associated with incorrect uptake. Counterevidence also depends on measurement: Galindo-Domínguez et al. (2026) reported opposing direct and indirect associations of self-regulatory variables with an outcome operationalized by one self-reported item on AI-use frequency. These results caution against equating fluent error, use frequency, or metacognitive bias with established overreliance or a single causal mechanism.

SAM therefore treats risk as testable transitions rather than a fixed causal chain: apparent credibility may reduce the perceived need to check; effort, access, or time constraints may discourage checking; and a check may fail to test the relevant claim. Repetition by the same system is not independent corroboration. Kaşarcı’s (2026) Epistemic Reliability Framework links output properties, vulnerability, epistemic risk, and scaffolding, overlapping with SAM in evaluation and support conditions. This evidence motivates SAM’s verification and epistemic-control hypotheses without establishing a joint causal effect of linguistic fluency and metacognitive calibration on overreliance. SAM’s resource distinctions and interaction predictions require incremental comparison. The review is a thematic synthesis, not a joint causal test. Its own sensitivity analysis (Kaşarcı, 2026, Table 5) identifies cognitive atrophy as one qualitative-study claim, no longer independently corroborated after that study’s exclusion. Immediate error uptake and longer-term developmental loss remain separate; proposition (P8) addresses the latter.

In a randomized writing study, ChatGPT improved essay revision scores and was associated with different self-regulated learning processes, without significant knowledge-gain or transfer advantages (Fan et al., 2025). In a randomized high-school mathematics trial, GPT Base improved supported practice but reduced subsequent unassisted performance relative to controls (Bastani et al., 2025). This provides no direct S measure, SAM validation, regulatory-mechanism test, or general long-term skill-loss evidence. These distinctions motivate separate assessment of supported products, independent performance, and development. This matters for AI-enabled education because judgment is both a competence learners must develop and one educators must exercise in guiding and evaluating learning. Since supported performance can improve without durable learning (Soderstrom & Bjork, 2015), SAM asks whether AI support preserves the evaluative and regulatory processes required for independent and accountable judgment.

Using clinical expertise as an example, Boshuizen and Schmidt (1992) show that expertise development involves the reorganization and compression of knowledge structures (“knowledge encapsulation”). The SAM analogy is that persistent substitution of integrative operations during acquisition may limit such development. This is not generative-AI evidence or proof of deskilling. Testing the analogy requires independent developmental comparisons; proposition (P8) specifies one for regulatory capacity.

SAM’s human-in-the-loop core proposes an asymmetric, testable division of labor, not an anti-automation rule: humans frame problems, calibrate delegation, and retain responsibility for validation and final judgment. Confirming a system-generated decision is insufficient. Architecture must enable evaluation through inspectable evidence, visible uncertainty, revisable suggestions, and contestable decisions. Without these affordances, absent checking cannot simply indicate low S or M; system design becomes a source of construct-irrelevant variance (Hollan et al., 2000; Lee & See, 2004).

A related task logic appears in a recent policy framework for AI-supported research: routine or labor-intensive activities (“labour”) may be accelerated by AI, whereas judgment-defining steps (“judgement”) should not be substituted by the system; AI may prepare or scaffold them (Zyphur, 2026). The broader point is that human-in-the-loop must be defined functionally in terms of who sets goals, evaluates uncertainty, selects evaluation criteria, revises results, and makes the accountable final judgment.

### 2.3. Regulatory Locus in Human–AI Interaction

Regulatory locus identifies where goal setting, monitoring, strategy adaptation, and reflection are initiated, performed, and revisable. The following arrangements describe task- and phase-specific allocations of regulatory functions, not fixed learner types, a mandatory developmental sequence, or scores of S.

First, human self-regulation is present when goals, monitoring, and regulatory decisions are initiated and controlled by the human. Second, AI-supported self-regulation can occur when the system provides cues, feedback, or scaffolds while regulatory authority remains with the human. Third, approaches to hybrid human–AI regulation describe configurations in which human and artificial intelligence make complementary contributions to regulation and mutually shape the process (Molenaar, 2022). Fourth, external or algorithmic regulation occurs when goal setting, monitoring, or strategy selection is taken over by the system to such an extent that human regulation is effectively reduced to confirming machine-prestructured options.

Co-regulation denotes relational regulation among humans; hybrid human–AI regulation denotes AI-supported or shared regulatory functions without treating AI as an intentionally equivalent social partner. AI can shape socially shared regulatory processes (Järvelä et al., 2023), while traditional co-regulation concerns human actors.

Self-regulation, co-regulation, shared regulation, and co-construction refer to different processes. In SAM, S denotes the individual capacity for self-regulation, distinguished from its enactment in a particular episode. Co-regulation and shared regulation describe relational forms of joint regulation, whereas co-construction denotes the joint generation, evaluation, or integration of knowledge (Fischer, 2002; Hadwin et al., 2017). Methodological competence concerns the individual capacity to select and apply methods for evaluating epistemic claims within such processes. Social processes thus remain distinct from SAM’s person-level resources.

S remains the individual’s regulatory capacity; regulatory locus describes its interactional conditions of exercise. External initiation of a prompt need not remove human control, whereas endorsing a system decision need not demonstrate it. Whether repeated arrangements change human capacity is the separate developmental hypothesis in P8.

Xu et al. (2025) provide a bounded illustration of external support in a GenAI setting. In a four-week study with 68 students in the final analysis, both groups used ChatGPT; one additionally received paper-based metacognitive prompts. Post-test group differences in SRL self-reports reached statistical significance for task strategy and self-evaluation, but not the other four dimensions; the achievement comparison was not statistically significant. This contrast concerns additional instructional support, not AI versus no AI or a test of regulatory-locus categories. Without a reported delayed assessment after removal of the target support, it does not establish durable regulatory-capacity gains or deskilling.

### 2.4. AI Literacy, AI Readiness, and SAM

**Figure 2:**
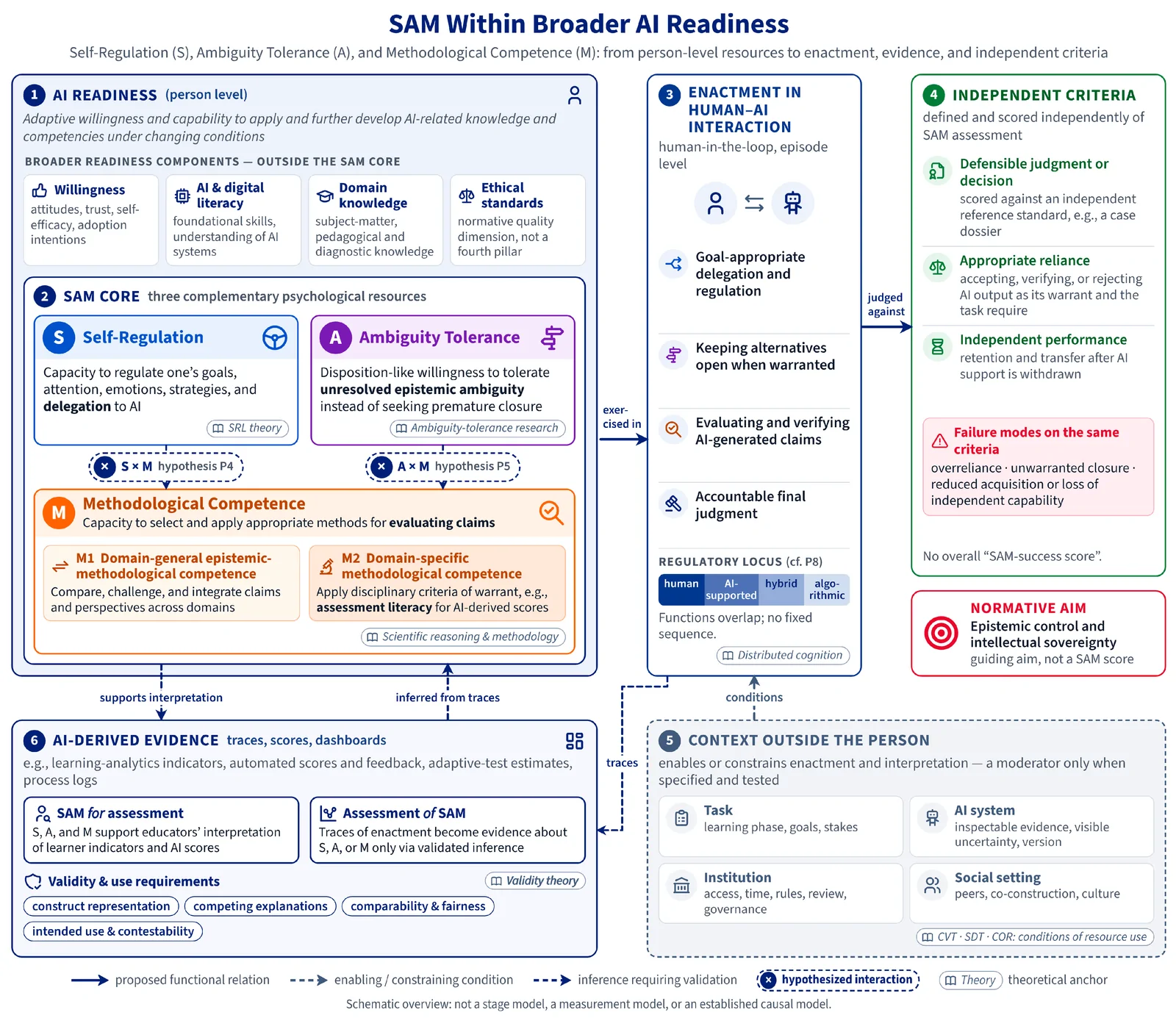
SAM within broader AI readiness. Six fields distinguish person-level readiness, the three SAM resources S, A, and M, enactment in human–AI interaction, independent criteria, external context, and AI-derived evidence. M1/M2 are a hypothesized differentiation within M. Context conditions enactment and interpretation; moderation requires specification and testing. Criteria are independent of resource assessment. Construct representation, competing explanations, comparability, fairness, and intended use constrain AI-derived evidence, not define outcomes. Integrated theoretical anchors support interpretation; Figure 3 specifies hypothesized resource–criterion relations. This is neither a stage model, a measurement model, nor an established causal model.

**Figure 3:**
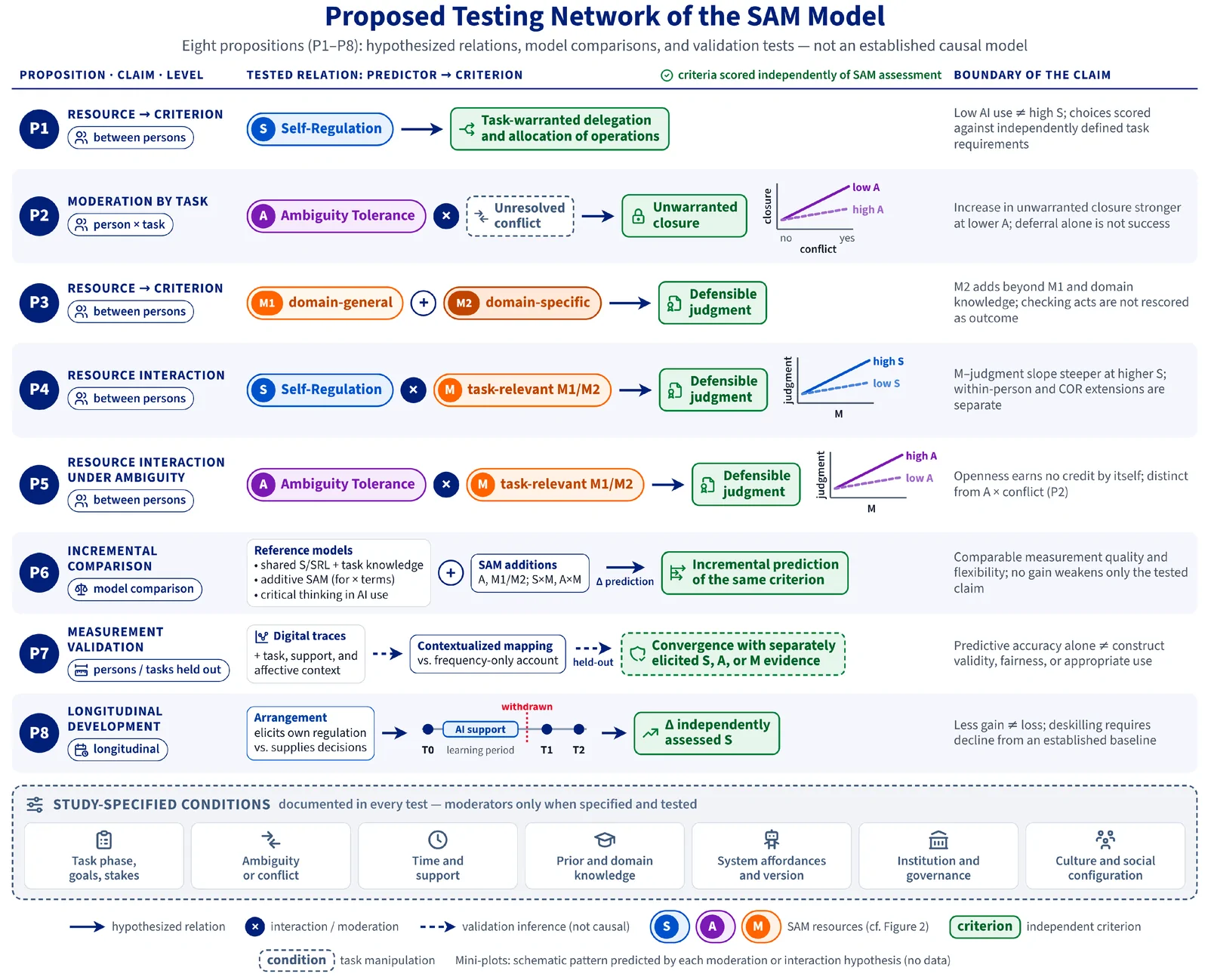
Proposed testing network of SAM. P1–P5 state distinct resource–criterion relations or interactions; P6 tests incremental prediction against shared and neighboring accounts; P7 concerns validation of indicator-to-resource interpretations rather than a causal path; and P8 concerns longitudinal effects of regulatory arrangements on independent regulation after target support is withdrawn. Contextual variables are study-specified conditions rather than automatic moderators. Criteria are defined independently of SAM resource scores, and arrows denote hypotheses rather than an established causal model. Mini-plots depict schematic predictions, not data.

AI literacy, AI readiness, and SAM overlap. AI literacy includes evaluation and collaboration (Long & Magerko, 2020); AI readiness includes cognition, ability, vision, and ethics (Wang et al., 2023). SAM’s bounded resource configuration must demonstrate added value against their actual content. Section 4.4 develops this comparison; Figure 2 and Figure 3 present the architecture and proposed relations.

For educator AI readiness, adoption, warranted use, and institutional opportunity are distinct questions. Attitudes, trust, self-efficacy, and usage intentions are broader readiness facets outside the SAM core; readiness measures may also include evaluative competence. Assessment literacy includes applying domain-specific criteria of warrant, a proposed expression of M2; data literacy also includes knowledge and prerequisites beyond M2. Their dispositions cannot generally be equated with A’s willingness to tolerate unresolved epistemic ambiguity. Strong individual resources may remain unexercised when access, time, or authority to challenge a recommendation is lacking. SAM complements institutional analysis and requires comparison with overlapping person-level measures; readiness does not guarantee its added value.

## 3. The SAM Framework: Three Complementary Psychological Resources

### 3.1. Functional Architecture and Anchor Case

SAM brings together person-level resources of different construct types, distinguished from their enactment in a particular interaction. S denotes the capacity to regulate one’s own goals, attention, emotions, strategies, and delegation. A denotes a disposition-like willingness to tolerate unresolved epistemic ambiguity. M denotes the learned capacity to select and apply appropriate methods of evaluation, with hypothesized domain-general (M1) and domain-specific (M2) components. This capacity is not merely knowledge of rules, but neither does possessing it imply exercising it in every episode. Observed performance provides evidence about a resource only through a justified inference; an omitted check establishes neither a lack of M nor the presence of unused M without additional evidence. Their proposed complementarity concerns distinct functions in epistemic control, not homogeneous traits, uncorrelated constructs, or exclusive behavioral markers. SAM assumes neither a single reflective higher-order factor nor a fixed sequence in which resources must be activated.

In the recurring anchor case, a preservice teacher uses a learning analytics dashboard and generative AI to interpret an unusual learning trajectory. Suppose the AI recommends remedial work because recorded activity is low, while comprehension difficulty and incomplete activity records remain plausible explanations. Willingness to tolerate this unresolved ambiguity (A) does not itself organize the next diagnostic step (S). Conversely, the teacher may find uncertainty aversive, yet regulate the urge for a quick answer and continue checking. M1 concerns her capacity to compare the explanations and identify evidence that would distinguish them; M2 concerns her capacity to evaluate what the dashboard measures and which inferences about understanding are warranted. Keeping both explanations open is not an exclusive marker of any one resource: it might also reflect confusion or compliance with an instruction. These hypothetical contrasts assume separate resource assessment before the target episode; they are not diagnostic rules. The case later distinguishes evidence about the teacher’s own SAM resources from the validity argument needed to act on the learner’s indicator.

### 3.2. Self-Regulation (S)

In the SAM model, self-regulation (S) denotes the capacity for goal-directed regulation of one’s own cognitive, metacognitive, motivational, emotional, and behavioral processes. This resource focus does not replace the process accounts of self-regulated learning (SRL). Panadero’s (2017) comparative review of SRL shows that major models converge on regulation across these domains while differing in their process architecture. Zimmerman (2000) emphasizes cyclical forethought, performance, and reflection, whereas Winne and Hadwin (1998) model regulation as recursive monitoring and control across task definition, goal setting and planning, enactment, and adaptation. Boekaerts (1999) adds the tension between learning goals and well-being, which is especially relevant when AI use changes perceived control or threat. Bandura’s (1997) self-efficacy framework complements these process models: perceived capability can shape choice, effort, and persistence but remains conceptually distinct from regulatory capacity and its exercise. Self-regulation strategies can be fostered through explicit instruction and structured learning environments (Dignath et al., 2008; Dignath & Büttner, 2008).

AI use requires ongoing decisions to delegate, verify, challenge, or work without support. Cognitive offloading (Risko & Gilbert, 2016) can reduce working memory demands and free resources for higher-order thinking. Its frequency is not the problem; unnoticed transfer of goals, monitoring, or a target operation to be learned can be. AI use is therefore an object as well as a means of self-regulation.

AI-supported work can alter perceived control over errors, authorship, and consequences while the value of fair or accurate judgment remains high. Section 4 specifies how control-value theory informs these affective conditions; the resulting emotions are distinct from the regulatory capacity needed to manage them.

In the anchor case, S is the capacity to maintain the diagnostic goal, organize checking, and revise delegation when support no longer serves that goal. It requires neither avoiding AI nor comfort with uncertainty. Relief, uncertainty, or frustration can affect its exercise without determining judgment.

The motivation to exercise regulatory capacity is distinct from that capacity. Section 4 relates self-endorsed versus controlled engagement to autonomy, competence, and relatedness without treating these as further SAM components.

For intervention, this logic can be operationalized as a Plan–Use–Check sequence. Before using AI, learners make the goal, their own contribution, and an initial hypothesis explicit; during use, they deliberately register delegation, revision, and, where relevant, affective warning signals; afterward, they justify what was adopted, changed, or rejected. The intended shift is toward learner control, with fading calibrated to the target operation and prior competence rather than withdrawal of every aid (Horz & Czora, 2026). Reading strategies for working with and reading AI-generated texts are one possible application of this logic (Brägger, 2026).

Delegation should be evaluated against the task goal and prior competence, not its frequency. During acquisition, assistance should leave opportunities to practice the target operation; in application, already-mastered routines may be offloaded while final judgment remains accountable. Intensive use and deliberate non-use can both be appropriate. These are task-sensitive design assumptions, not evidence that observed compliance demonstrates S or that assistance necessarily improves learning.

### 3.3. Ambiguity Tolerance (A)

Classic work on intolerance of ambiguity (Budner, 1962) and integrative accounts of tolerance of uncertainty (Hillen et al., 2017) underscore the importance of distinguishing related but nonidentical forms of epistemic indeterminacy. Ambiguity, risk, uncertainty, and ignorance must be distinguished analytically. Risk describes situations in which outcomes and probabilities can in principle be specified; uncertainty involves limited or not reliably quantifiable information; ignorance denotes missing relevant knowledge; ambiguity arises when several interpretations, models, or epistemic claims remain simultaneously plausible. Within SAM, A denotes a disposition-like willingness to tolerate unresolved epistemic ambiguity rather than seek closure merely to end discomfort. It is neither emotion-regulation capacity nor resilience, and it is not equivalent to low anxiety. Tolerating ambiguity can involve discomfort; regulating the emotions it elicits is primarily a function of S. This matters under bounded rationality because complete information and unlimited processing capacity are rarely available (Gigerenzer & Gaissmaier, 2011; Simon, 1955).

AI-supported tasks can present conflicting interpretations, incomplete evidence, or limited explanations. These conditions may elicit epistemic emotions, but neither the uncertainty of the task nor the emotional response is A. Section 4 distinguishes the appraisal-based account of these emotions from willingness to tolerate unresolved ambiguity.

S and A are distinguished by function, not by a fixed temporal sequence. A concerns willingness to tolerate unresolved ambiguity; S concerns the capacity to organize and adjust attention, emotion, goals, strategies, and delegation. M concerns the capacity to evaluate competing claims against appropriate criteria. Their exercise can overlap within an episode. Keeping alternatives open, delaying a decision, or reaching a rapid conclusion does not identify any one resource without further evidence.

Social interaction can expose competing interpretations and interests through perspective taking, negotiation, and co-construction. Research on knowledge integration links perspective taking, reflexivity, and tolerance of ambiguity and uncertainty with successful integration of heterogeneous knowledge (Vogel & Hunecke, 2024); models of social uncertainty describe perspective taking as a controlled inferential process when automatic interpretations are insufficient (FeldmanHall & Shenhav, 2019). Perspective taking is not A, and disagreement need not leave a claim genuinely ambiguous. Proposition P2 specifies A-related differences in responses to conflict; proposition P5 concerns A × M within ambiguous tasks. Social integration remains an interaction process, not an individual resource score. Behavioral tolerance of ambiguous probabilities has been associated with cooperation and trust (Vives & FeldmanHall, 2018); its equivalence to SAM’s broader A construct and the causal effects of repeated negotiation on A remain to be tested.

Cultural influences should be treated as contextual conditions, not as an individual-level definition of A. Whether cultural norms moderate the expression, evaluation, or measurement of keeping options open, dissent, or readiness to decide must be specified and tested. Hofstede’s (2011) “uncertainty avoidance” is a culture-level aggregate and must not be equated with individual ambiguity tolerance. Cross-cultural claims about A accordingly require measurement invariance and context-sensitive validity arguments.

In the anchor case, willingness to keep both explanations open is distinct from mistrust of the dashboard. The relevant uncertainty concerns what the recorded activity warrants, not whether AI should be trusted in general.

A is not itself a criterion of decision quality. Neither prolonged openness nor rapid closure is inherently preferable: a decision or deferral must be justified by the available evidence and task demands. Tolerance of ambiguity and warranted judgment therefore require distinct assessment questions.

### 3.4. Methodological Competence (M)

Methodological competence (M) is the learned capacity to select and apply domain- and methodologically appropriate procedures for evaluating epistemic claims. It draws on declarative, procedural, and conditional methodological knowledge but is not reducible to knowing rules. The construct concerns what a person can do under specified task conditions; performance in a particular interaction supplies evidence for that capacity rather than defining it by occurrence. M encompasses judging which data, sources, measurements, arguments, perspectives, or tests can support, refute, or delimit a claim. In SAM, M is differentiated as a theoretical structural hypothesis into two related components: domain-general epistemic-methodological competence (M1) and domain-specific methodological competence (M2). Empirical support for this distinction comes from Schunn and Anderson (1999): experts from different psychological subdisciplines showed advantages in general scientific reasoning skills, whereas memory researchers additionally excelled in domain-specific experimental design. This pattern supports distinguishing transferable methodological procedures from domain-bound methodological expertise, while leaving SAM’s proposed M1–M2 structure to be tested directly. The measurement model for M1 remains open. Reflective, formative, and multidimensional representations make different assumptions about the relation between competence and its indicators; these assumptions must be justified theoretically before their empirical adequacy is compared. Statistical fit alone cannot decide among them.

#### 3.4.1. M1—Domain-General Epistemic-Methodological Competence

M1 refers to the capacity to use epistemic-methodological practices across domains. Their common function is to coordinate, compare, challenge, and integrate competing epistemic contributions; some of these practices are inherently social-epistemic, but the construct is defined by this evaluative function rather than by the presence of social interaction. M1 includes practices for clarifying epistemic problems and goals and for systematically comparing alternatives. It also encompasses social–epistemic practices such as perspective taking, argumentative justification, counterargument, negotiation of meaning, and the establishment of common ground. A third group concerns the integration and critical examination of evidence, including the coordination of different knowledge contributions, co-construction of shared explanations, cross-domain comparison of multiple sources and forms of evidence, generation of counterexamples, and reflection on one’s own and others’ epistemic positions. M1 denotes neither general sociability nor a personality trait, but a learnable capacity to generate, evaluate, and integrate knowledge, including through joint epistemic work.

Research on computer-supported collaborative learning (CSCL) provides a conceptual basis for this component. Fischer (2002), for example, analyzes collaborative knowledge construction in terms of argumentative discourse and collective information processing; collaboration scripts structure interaction so that activities relevant to knowledge construction become more likely (Kollar et al., 2006). Kollar et al. (2007) found that more structured external scripts supported domain-general argumentation knowledge, whereas domain-specific knowledge gains were associated with learners’ internal scripts; the external-script manipulation did not improve the overall domain-specific knowledge measure. This does not validate the SAM distinction between M1 and M2, but it provides a relevant conceptual connection for the hypothesis that domain-general epistemic practices and domain-specific criteria of warrant may be analytically distinguishable.

Collaboration is a social situation and co-construction an interaction process. M1 concerns individual capacities for epistemically reconstructing others’ contributions, requesting reasons, relating arguments, identifying contradictions, establishing shared frames, weighting evidence, and revising explanations. Section 3.5 summarizes its relation to S and A.

#### 3.4.2. M2—Domain-Specific Methodological Competence

M2 is the capacity to apply the procedures and criteria of warrant within a particular epistemic domain. In quantitative–empirical contexts, these include measurement, validity, statistical uncertainty, sampling, and causality; in the natural sciences, experiment, measurement error, boundary conditions, and replication. In qualitative and interpretive contexts, transparency of sampling and interpretation, reflexivity, plausibility, triangulation, and consideration of alternative interpretations are central quality concerns (Tracy, 2010). In historical and humanities contexts, disciplinary evaluation involves source provenance, authorship, context of production, corroboration, textual evidence, perspectivity, and competing interpretations. Wineburg’s (1991) analyses of historical reading illustrate how strongly expertise here depends on a specific epistemological stance toward sources. In mathematics and computer science, relevant practices include proof, boundary cases, counterexamples, testing, and transferability. M2 is therefore necessarily situated within disciplines without abandoning SAM’s general principle of methodologically warranted evaluation.

At the M1 level, triangulation refers to the cross-domain practice of comparing and coordinating multiple sources or perspectives. At the M2 level, it means applying domain-specific criteria to decide which forms of evidence can be combined meaningfully and what inferences that combination warrants.

The M1/M2 distinction draws a structural analogy to professional-competence models separating general educational–psychological from subject-matter and pedagogical-content knowledge (Baumert & Kunter, 2013). Their model supplies structural inspiration, not a theory of SAM’s methodological competence.

#### 3.4.3. Interrelations Between Domain-General (M1) and Domain-Specific Methodological Competence (M2)

M distinguishes rhetorical or intuitive plausibility from supporting evidence. Research on statistical literacy, scientific reasoning, and epistemic vigilance shows that professionally convincing information can be misinterpreted without evaluative competence (Gigerenzer et al., 2007; Schmidt et al., 2021; Sperber et al., 2010). This includes quantitative civic claims (Gigerenzer, 2022).

Gigerenzer’s (2018) critique of “statistical rituals” sharpens this point: methodological competence must not be confused with mechanical rule following. Formal plausibility cues cannot replace examination of evidence and inference (Isberner et al., 2013). Section 2.2 applies this distinction to AI-output uptake without equating performed checking with successful validation.

M supplies evaluation and integration criteria. M1 supports structured negotiation, perspective integration, and domain-general evaluation; M2 translates outputs and shared interpretations into disciplinary warrant questions. Their cross-domain core is testing AI-generated or co-constructed plausibility against explicit reasoning and evidence standards, not identical methods.

In the anchor case, comparing the two explanations draws on M1, whereas evaluating the dashboard’s measurement basis and the limits of a competence attribution draws on M2. A reasoned acceptance, qualification, or rejection can provide performance evidence, but neither a correct conclusion nor the occurrence of checking identifies M independently of regulatory and contextual conditions.

In scientific research, M2 can be specified particularly concretely. The Instats policy report on Responsible AI in Academic Research explicitly frames AI literacy at the PhD level as a problem of judgment rather than tool use and places validity reasoning at its center (Zyphur, 2026). Recommended practices include checking citations against primary sources, documenting model versions and relevant parameters, testing sensitivity to prompt variation, conducting adversarial evaluation using sufficiently heterogeneous models, and detecting sycophancy. These recommendations should be understood as a domain-specific policy framework, not as psychometric validation of SAM. They illustrate how the general M question—what justifies a claim, and what evidence would falsify it?—can be translated into a concrete epistemic practice.

Possessing M does not ensure its exercise, which requires time, attention, and inspectable evidence. Section 4’s resource-investment account treats these as conditions of evaluative activity, distinct from M and the S × M prediction.

### 3.5. Construct Types, Functional Boundaries, and Instructional Proximity

The proposed complementarity concerns distinct functions rather than exclusive behavioral markers: S concerns regulation of epistemic activity, A willingness to tolerate unresolved ambiguity, and M the ability to evaluate claims. Interaction and compensability claims go beyond this functional distinction and require separate empirical tests.

#### 3.5.1. Measurement Implications and Limited Compensability

SAM does not require a unidimensional or hierarchically reflective measurement model. An additive model of correlated resources provides the reference for the between-person S × M and A × M hypotheses in Section 6. Tests must specify the relevant M component and use independent outcome criteria (Section 5.4); no M total score is assumed. The configurational claim requires predictive gain beyond the additive model, not merely better performance by people high on both resources. Limited compensability is a further claim: within a pre-specified task and resource range, can stronger other resources fully offset a specified weakness? Outcomes, profiles, and the contrast counting as compensation must be defined in advance. Evidence of full compensation would weaken the limited-compensability claim even if an interaction were present. An interaction establishes neither universal thresholds nor necessity.

#### 3.5.2. Differences in Instructional Proximity and Malleability

S, A, and M differ in construct type and directness of change through instruction and experience. This proposed gradient of instructional proximity is more informative for intervention and measurement than treating them as equally teachable.

As a dynamic regulatory resource, S is relatively close to instruction: strategy instruction, modeling, feedback, scaffolding, and gradual fading can foster self-regulatory processes from an early stage (Dignath et al., 2008; Dignath & Veenman, 2021). A, by contrast, has a stronger dispositional and affective component. We therefore expect ambiguity tolerance to respond less directly to instruction than a specific strategy, although this comparative claim requires intervention evidence and does not imply an immutable personality characteristic. Learning environments can repeatedly expose learners to limited information, probabilistic or conflicting evidence, and decisions under uncertainty.

M is more instruction-proximal because evaluation knowledge, procedures, and practices can be modeled, practiced, and given feedback. M1 examples include comparing perspectives, constructing arguments and counterarguments, and integrating evidence; M2 examples include disciplinary source evaluation, counterexamples, boundary cases, and other validation procedures.

## 4. Why These Three Constructs? Functional Distinctiveness and Boundary Conditions

The boundary of the SAM core is as important as its contents. Social, ethical, digital, motivational, and institutional factors are consequential for AI-enabled education, but they need not be modeled as additional intraindividual resources.

The proposed selection is functional, not exhaustive (Section 1.2). S concerns regulatory capacity, A willingness to tolerate unresolved ambiguity, and M the capacity for methodological evaluation; whether these functions require the proposed differentiation remains open. SRL already integrates cognitive, metacognitive, motivational, affective, and behavioral aspects (Panadero, 2017). Metacognitive monitoring and control are therefore not new SAM functions. Epistemic vigilance addresses mechanisms for dealing with potentially misleading communication (Sperber et al., 2010), overlapping the scrutiny of claims. Hybrid human–AI regulation addresses the distribution and transfer of regulatory control (Molenaar, 2022); SAM proposes person-level resource hypotheses within such arrangements rather than replacing them. These neighbors are not designated antecedents or subordinate mechanisms by definition. The explanatory proposal concerns resource complementarity and the conditions of exercise; its empirical contribution requires the discriminant and comparative tests in Section 5 and Section 6. Alternative decompositions remain admissible.

Theoretical accounts serve distinct roles. SRL provides a theoretical foundation for S; A and M have construct-specific foundations (Section 3). Distributed cognition and joint cognitive systems describe work across people and artifacts, not additional person-level resources. Affective, motivational, and resource-investment accounts explain exercise conditions, not SAM’s structure. Validity theory governs assessment and score-use inferences. Applying assessment-literacy warrant criteria is a proposed expression of M2 when educators interpret AI-derived evidence. Institutional affordances and ethical responsibility remain contextual and normative, outside the intraindividual core.

Control-value theory (CVT) links perceived control and subjective value to achievement emotions (Pekrun, 2006), extending to epistemic and social emotions (Pekrun, 2024). AI-task appraisals of authorship, errors, fairness, or consequences help explain resource-exercise conditions; appraisals are not themselves S or A. Similar discomfort can accompany different regulation; emotion determines neither ethical correctness nor warranted judgment.

Self-determination theory (SDT) distinguishes autonomous and controlled motivation and autonomy, competence, and relatedness support (Deci & Ryan, 2000; Ryan & Deci, 2020). It motivates hypotheses about sustained, self-endorsed use of regulatory and methodological capacities. AI or institutional requirements can change professional discretion; better supported products are not independent competence. Relatedness concerns human relationships in learning and professional judgment. These conditions are neither additional SAM resources nor evidence of increased M.

Conservation of resources (COR) theory addresses retaining, protecting, and building valued resources and investment under threatened or actual loss (Hobfoll, 1989; Hobfoll et al., 2018). SAM asks whether constraints reduce evaluation investment despite available application capacity. Available time and support, perceived scarcity, and effort remain distinct. This is separate from between-person S × M; time pressure alone identifies no COR mechanism. Resources, investment, and contextual effect direction require specification. CVT, SDT, and COR motivate conditions, not automatic covariates, moderators, or mediators.

### 4.1. Social Interaction and Co-Construction as Developmental, Methodological, and Interactional Conditions of SAM

Social interaction is not a fourth SAM resource. It can expose and activate resources, provide perspective diversity and disagreement for development, generate ambiguity-relevant epistemic heterogeneity, and distribute regulation. Collaboration is a relational process whose quality depends on individual and interactional conditions (Fischer, 2002; Hadwin et al., 2017; Järvelä et al., 2023; Vogel & Hunecke, 2024).

Human co-construction brings intentional positions, social responsibility, and potentially reciprocal perspective taking together. Generative AI can supply contributions, counterarguments, perspectives, and questions that stimulate or simulate co-construction without ontological symmetry. We therefore use AI-supported co-construction and human–AI-supported knowledge construction cautiously; ultimate epistemic responsibility remains human.

The dialogic and responsive surface of generative AI makes this distinction psychologically consequential. Classic research shows that people apply social rules, reciprocity, and other social expectations to computers (Nass & Moon, 2000; Reeves & Nass, 1996). More recent work describes parasocial relationships with generative AI chatbots and examines the conditions under which users experience such systems as socially meaningful interaction partners (Zhong & Luo, 2026). In the model, AI may be perceived as a socially meaningful, quasi-social, or parasocial interaction partner, but it is not assigned the same intentional agency as a human. Social or anthropomorphic cues may alter trust, reliance, willingness to challenge, perspective taking, negotiation behavior, and cognitive closure and should therefore be examined as candidate conditions for the exercise of S, A, and M; moderation requires explicit specification and testing.

### 4.2. Ethical Reflection: A Normative Quality Dimension, Not a Fourth Pillar

Ethical reflection is an indispensable quality dimension of responsible AI use, but not an independent psychological pillar of SAM. Normative questions concern S-related goal and delegation decisions as well as M-related evaluation of bias, validity limits, authorship, and potential harms. The control-value perspective helps explain why such questions can become emotionally salient: when authenticity, fairness, or professional responsibility is highly valued while control over the origin or consequences of an AI contribution is uncertain, guilt, shame, concern, anger, or mistrust are plausible responses (Pekrun, 2024). The emotion itself does not determine what is ethically right; it may signal a need for evaluation that must then be regulated and addressed methodologically or normatively.

### 4.3. Foundational Skills Within Broader Digital and AI Literacy

Foundational computer knowledge and skills can enable access and informed use. The computer-literacy inventory distinguishes practical and theoretical knowledge from computer anxiety and attitudes (Richter et al., 2010). AI literacy encompasses evaluation and collaboration alongside understanding of data and system limitations (Long & Magerko, 2020). SAM therefore distinguishes enabling skills from its focal resource functions without reducing either literacy framework to technical operation.

Digital-skills frameworks include critical thinking and problem solving (van Laar et al., 2017). M specifies the capacity to apply appropriate standards to a claim without denying evaluation in literacy. Overlap with literacy measures requires Empirical Tests of the Model Section’s comparisons. Defining resources by functions rather than interfaces is a modeling choice; generalizability under system change remains untested.

### 4.4. SAM in Relation to Current Models of Learning and Working with AI

Several complementary frameworks focus on instructional, professional, or organizational questions. Döbeli Honegger (2025) and Kerres et al. (2025) address changes in media and information practices; Brägger and Rolff (2025) and Fullan (2025) address instructional, school, and system development; and Buck and Weßels (2025) address leadership and the division of labor with AI. The technological pedagogical content knowledge (TPACK) framework (Mishra & Koehler, 2006) specifies profession-specific integrative knowledge. These approaches are not necessarily competing models; their relevance depends on the research question. SAM overlaps with AI-literacy and AI-readiness frameworks (Long & Magerko, 2020; Wang et al., 2023; Section 2.4) where they address evaluation but adds no further competencies or dimensions; within this broader readiness, it specifies the person-level resources hypothesized to condition whether claims arising in human–AI interaction are actually evaluated against appropriate criteria of warrant, and how these resources interact.

Critical thinking in AI use provides a closer person-level comparator. Lau et al. (2026) distinguish Verification, Motivation, and Reflection in a 13-item self-report scale; higher scores were associated with more frequent and diverse self-reported verification and, separately, more accurate veracity judgments in an AI-mediated fact-checking task. Reported frequency and diversity do not establish verification quality or M. Its content concerns checking source and content, understanding AI, and reflecting on responsible use. These functions overlap with SAM without establishing equivalence to S, A, and M. Different labels or assessment methods do not establish distinct resources. SAM’s proposed addition is the bounded configuration and its separately testable resource and interaction contributions, not critical evaluation itself. Empirical Tests of the Model Section specifies tests against this account. A complementary contextual hypothesis—that didactic and organizational provisions support warranted judgment more when relevant resources are stronger—remains untested and implies no superiority over neighboring models.

### 4.5. SAM Within Broader AI Readiness: Architecture and Boundaries

Figure 2 presents SAM’s architecture and boundaries within broader AI readiness. It separates person-level resources from their overlapping enactment, contextual conditions, theoretical roles, and independently defined criterion families; it does not depict a fixed process sequence. Figure 3, then, maps the eight propositions onto distinct resource, criterion, comparator, measurement, and developmental claims.

## 5. Assessment of SAM

### 5.1. From Observable Traces to Psychological Constructs: The Inferential Problem

Prompts, revisions, source access, and acceptance of suggestions can inform assessment only relative to the task and the intended resource interpretation. No recorded action is itself S, A, or M. Theory-linked inferences require other methods and tests of alternative explanations (Boulahmel et al., 2025; Fan et al., 2022).

“Assessment of SAM” concerns S, A, and M construct representation in AI-based learning and assessment. “SAM for assessment” concerns human resources for interpreting other AI-derived indicators, including engagement, competence, or risk. This separates resource measurement from professional reasoning quality in decisions based on automated measurement.

In the dashboard case, consider assessing the preservice teacher’s assessment-related M2 from how she inspects source records. Merely opening them could reflect an instruction, confusion, or interface availability. A candidate interpretation instead links the inspected evidence to her explanation of what the dashboard measures and what can be inferred about comprehension. Test this interpretation against separately elicited measurement-evaluation performance across cases, documenting prior knowledge, language demands, and support. Agreement across methods under contrasts designed to distinguish these explanations would support the interpretation; systematic discrepancies would challenge it. Later prediction of judgment quality must use a different dossier and independent scoring (Section 5.4). Such evidence concerns the teacher’s resource, not the validity of the dashboard’s learner score. Diagnostic use requires the additional safeguards in Section 5.5.

Validity concerns inference from behavior to a specified resource and its use, not the log variable (Embretson, 1983; Kane, 2013; Messick, 1995). The argument must address construct representation and alternative causes under task/system conditions: identical actions can support different interpretations until their inferential links are independently tested.

The relation between trace and construct is many-to-many. Lazarides et al. (2026) illustrate this problem through machine learning (ML) classification of teachers’ nonverbal immediacy. The measure samples selected features of a broader relational construct, and the authors identify instructional phase as an untested alternative explanation for observed relations. The study thus informs SAM’s demands on construct coverage and contextual interpretation; it does not validate SAM indicators or establish that instructional phase caused the observed associations.

A positive precedent is provided by Gupta et al. (2024): in Crystal Island, models combined game traces, prior-knowledge scores, and earlier written reflections to predict science post-test scores and subsequent reflection ratings, evaluated on held-out students. They also examined prediction disparities by gender and prior gaming experience and reported trade-offs after bias mitigation. This illustrates criterion-linked prediction and separate fairness testing, not direct measurement of SAM resources. Whether using these predictions to deliver support improves learning remains a further intervention question.

### 5.2. Construct Representation, Underrepresentation, and Construct-Irrelevant Variance

Two threats are especially important. Construct underrepresentation occurs when an indicator samples only a narrow part of a resource; construct-irrelevant variance arises when behavior depends on language proficiency, typing speed, prior knowledge, interface experience, access, or system characteristics rather than the target resource (American Educational Research Association et al., 2014; Messick, 1995). System affordances are part of this specification: if users cannot inspect sources, access information about uncertainty, revise recommendations, or challenge decisions, a lack of checking cannot be attributed simply to low S or M (Hollan et al., 2000; Lee & See, 2004). Validation should therefore document what actions the environment makes possible and test competing interpretations of the observed pattern.

### 5.3. Measurement Approaches for S, A, and M

Assessing regulatory capacity in AI-assisted environments requires an explicit argument linking observations to the intended resource interpretation; the following paragraphs specify these inferential requirements. Assessing S as regulatory capacity requires distinguishing self-appraisal, situated enactment, and the inference from performance to capacity. Self-report instruments such as the Motivated Strategies for Learning Questionnaire (MSLQ; Pintrich et al., 1993) assess reported motivation and learning strategies; they do not directly measure regulatory capacity. Their contribution depends on the scales selected and their task and time referents. They need not reproduce the processes observed in a particular AI interaction to provide useful evidence. Measurement must match the temporal and functional level of the intended interpretation (Winne & Perry, 2000); a between-person resource claim cannot be established by relabeling a single episode’s regulatory activity.

Event-proximal methods address the separate question of how regulation is enacted. Experience sampling can capture thoughts, affective states, and regulatory decisions close to the action and reduce retrospective reconstruction (Zirkel et al., 2015). Perceived control, subjective value, discrete emotions, and regulatory action should be assessed separately. The Epistemically Related Emotion Scales, for example, assess surprise, curiosity, enjoyment, confusion, anxiety, frustration, and boredom during epistemic activities (Pekrun et al., 2017). Think-aloud protocols, situational-judgment formats, and performance tasks provide complementary evidence about reasoning and choices. These observations can inform the process account without being treated as interchangeable measures of S or A.

Qualitative work also illustrates why process categories should not be treated as psychometric dimensions. Dai (2026) identified planning, monitoring, evaluating, information-management, and debugging strategies among 12 postgraduate L2 students using ChatGPT, based on interviews and retrospective reflections with chat logs as supplementary evidence. The study informs candidate process categories for S-related enactment but does not validate a measurement model or map those categories onto SAM resources.

Models of regulation guide the interpretation of traces (Winne, 2011; Winne & Perry, 2000), with task context and evidence from other methods needed to evaluate competing explanations (Boulahmel et al., 2025; Fan et al., 2022). Winter et al. (2024) illustrate engagement-related trace information, not a direct measure of S. A candidate S assessment could compare goal-relevant planning and adaptation across tasks with documented delegation demands and support. Reasons for choices and prior knowledge would help distinguish regulatory adjustment from trial and error or compliance. Such comparisons test a proposed capacity interpretation; simply aggregating prompt changes or revision counts across episodes does not establish it.

McLain’s (2009) Multiple Stimulus Types Ambiguity Tolerance Scale-II (MSTAT-II) assesses orientation toward complex, unfamiliar, and insoluble stimuli. It provides a candidate dispositional measure, but its fit to SAM’s specifically epistemic interpretation of A must be tested rather than assumed. Validation should examine whether this interpretation can be distinguished from anxiety, confusion, decision slowness, and limited domain knowledge.

A candidate design would combine dispositional self-reports with contrastive vignettes varying interpretive ambiguity, documenting the information and options available. Perceived ambiguity, willingness to tolerate it, emotional responses, and reasons for closure should be recorded separately. Consistency across occasions and methods would be examined alongside competing explanations, not presumed from repeated observation. Control-value appraisals and emotions may explain the expression of A, but their causal and statistical roles require specification rather than automatic adjustment. Valentin and White (2026) report associations between intolerance of uncertainty and more avoidant and less rational self-reported decision-making styles; this concerns a neighboring construct, not AI-specific validation of A. Predictive tests must use independently justified judgment criteria rather than reward continued openness itself.

To assess M as a capacity, tasks should elicit evaluation rather than only recognition or recall of methodological rules. Candidate M1 tasks include collaborative problem solving, structured controversy, perspective integration, argumentation, and situational judgment; scoring can examine justification, counterargument, common ground, and the integration of heterogeneous evidence. M2 tasks require domain-specific source criticism, statistical or experimental validation, historical or interpretive evaluation, and counterexample or boundary-case tests. Erroneous, contradictory, or incomplete AI outputs can provide material for either component, provided the task demands distinguish the intended functions. Knowledge tests and self-reports contribute evidence about prerequisites and self-appraisals, not sufficient evidence of application capacity on their own.

Performance-based assessment does not make M directly observable. The inference concerns evaluative capacity under specified conditions, not performance in an isolated AI interaction. It must address contributions of regulation, ambiguity tolerance, domain knowledge, and opportunities to inspect and challenge outputs. Individual M1 inferences from collaborative tasks additionally require attributable contributions and separation of partner and group effects, including conformity. Comparisons across tasks or systematically varied support can test competing interpretations; supportive conditions do not produce a pure measure of M. Evidence for M1/M2 differentiation must come from the pattern across appropriate tasks, not their labels alone. In predictive tests, resource assessment and outcome must be distinguished in definition, elicitation, and scoring: success is judged against independently specified task standards, not by rescoring the same methodological actions.

In educational settings, portfolios and case-based assessments can additionally be scored with analytic rubrics that emphasize the methodological path rather than the final product alone (Ehlers, 2020). The recent review literature on digital literacy identifies Likert-based instruments, framework-oriented tools, and performance-based measures, but also points to the dominance of self-report, limited cross-cultural validation, and a lack of authentic performance formats (Suri et al., 2025). For SAM, portfolios provide candidate performance evidence about M under the interpretive requirements above. Table 1 separates target resources from possible observations, contextual requirements, and validity threats.

### 5.4. Multimethod Assessment and Independent Outcome Criteria

Multimethod assessment should test the proposed interpretation, not merely increase the number of data channels. Self-reports concern appraisals and reported tendencies; traces document activity; situational-judgment and performance tasks elicit choices and application. For S, repeated revision could reflect goal-directed adaptation, difficulty, or compliance. Linking revisions to task goals, reasons, prior knowledge, and support conditions can help distinguish these explanations; a frequency score alone cannot. Predicted convergence and informative divergence should be specified using construct-appropriate methods and measurement models that address measurement error and shared task or method effects. Table 2 assigns methods complementary roles; independently justified outcome criteria remain separate from evidence used to assess resources.

Outcome assessment serves a different purpose from resource assessment. Successful performance is judged against task requirements and domain-specific reference standards specified independently of SAM scores. Acceptable conclusions, evidential limits, and warranted nondecisions must be defined from the information available to the participant; neither inaccessible case truth nor agreement with an AI output is a sufficient standard. Resource and outcome tasks must be separated in definition, elicitation, and scoring, and outcome ratings should not be informed by resource scores. A correct answer does not by itself demonstrate epistemic control; process evidence addresses the person’s role in reaching it, not an alternative scoring rule that equates success with SAM-defined actions.

In the dashboard case, an independently prepared reference dossier could specify which conclusions about comprehension the available records warrant. Resource evidence would be elicited beforehand on separate tasks or cases. Outcome raters unaware of SAM scores would score judgments on a different dossier using independently specified rules, domain standards, and evidence available to participants, allowing multiple defensible decisions where warranted. Task separation does not establish discriminant validity. Where records are incomplete, qualifying a competence attribution may be appropriate; where convergent evidence resolves the issue, continued deferral need not be. Task success would be the defensibility of the resulting judgment or recommendation, not the number of checks or time spent deliberating. Contrastive cases could assess whether relevant evidence changes produce warranted judgment adjustments. Appropriate reliance can then be evaluated relative to the recommendation’s warrant and task demands. The quality of checking procedures may inform resource or process assessment, but must not simply be reused as an independent validation-quality outcome.

A separate criterion family concerns independent performance, retention, and transfer after the target AI support is withdrawn. Tasks, other available support, and time intervals must be specified; claims of learning, maintenance, or loss require an appropriate baseline or comparison. Studies should select criteria suited to their claims rather than combine these families into a universal SAM-success score. Reference standards and scoring remain subject to validation, particularly where several judgments are defensible or experts disagree.

### 5.5. Fairness, Measurement Invariance, and Consequences of Interpretation

Validation must distinguish predicting a criterion from interpreting a score as a SAM resource. Measurement comparability concerns whether that interpretation is sufficiently similar across groups, contexts, and system versions; algorithmic fairness concerns justified group-sensitive standards for prediction errors or decisions. Neither establishes the other. Examine relevant access, language, disability, cultural, and system conditions, and uncertainty in subgroup estimates (American Educational Research Association et al., 2014). Before diagnostic deployment, specify the intended decision, likely consequences, review and correction procedures, and what evidence permits that use. An exploratory indicator might justify a question to the learner without warranting a deficit label or high-stakes allocation. Support triggered by an indicator may change subsequent behavior; its effects require evaluation separately from the initial measurement claim.

An AI-specific stability problem arises because the measurement environment changes with the system. New models can alter response quality, access to sources, latency, agentic functions, or the difficulty of meaningful control; the same user behavior may then acquire a different psychological meaning. We refer to this problem as measurement drift under system change. Measurement invariance should therefore be examined not only across groups but also across tool generations and task ecologies. Lazarides et al. (2026) provide a useful operational precedent from ML-based classroom measurement: video segments were split into training and validation sets at the teacher level so that no teacher appeared in both sets, while the two sets also differed in contextual recording conditions such as classroom layout, camera placement, and lighting. This does not establish invariance across AI-system versions, but it illustrates the relevant validation logic: person-related signal should be tested under nonidentical contextual conditions rather than allowing recording or system conditions to leak into the construct interpretation. System version and task ecology consequently belong in the measurement specification, and longitudinal analyses must distinguish competence change from change in the interaction architecture. Robust SAM assessments should be tied to functional classes of behavior rather than proprietary interface metrics.

## 6. Nomological Network and Empirically Testable Propositions

The nomological account distinguishes enabling and contextual conditions, person-level resources, their exercise in human–AI interaction, and independently defined outcomes (Section 5.4). Figure 2 presents the architecture and Figure 3 depicts the testing network; neither establishes a causal sequence. The contextual accounts in Section 4 concern conditions of resource use, including COR-related investment; they are not additional resources or part of the S × M term. Feedback may affect later resource use or development, but changed behavior alone does not demonstrate changed capacity.

From the model’s construct definitions, interaction logic, measurement argument, and regulatory-locus account, we derive eight empirically testable propositions. P1–P6 concern resource contributions and their relations to performance; P7 concerns measurement. P8 asks whether repeated regulatory arrangements change human capacity.

**Proposition 1 (*Self-Regulation and Goal-Appropriate Delegation*).**  *Under specified task and support conditions, higher pre-assessed S should predict delegation decisions that better meet independently defined task requirements (Section 5.4), given prior competence and the information available at the decision. Appropriateness concerns which operations are retained or delegated and how their allocation serves the stated goal, not minimal AI use or the frequency of regulatory actions. In the dashboard case, delegating a routine summary may be appropriate while selecting the diagnostic method remains a learning target for the preservice teacher. That allocation must be justified by the task, not imposed as a universal rule. P1 concerns between-person resource differences; the S × M prediction in P4 is a separate claim*.

**Proposition 2 (*A as a Moderator of Conflict-Related Unwarranted Closure*).**  *Compared with otherwise comparable tasks in which evidence permits a determinate judgment, conflicting claims that leave the judgment unresolved are predicted to increase unwarranted closure more strongly at lower than at higher pre-assessed A. Closure is unwarranted when a commitment exceeds what the available evidence and independently defined task standards (Section 5.4) support, not merely when it is rapid. Deferral is not inherently successful. This A × conflict prediction differs from A × M within ambiguous tasks. Appraisals and emotions may help explain responses but are neither A nor automatically established mediators*.

**Proposition 3 (*Methodological Competence and Independently Assessed Judgment Quality*).**  *Under specified task and support conditions, higher methodological capacity is expected to predict more defensible judgments of AI-supported claims, using the independent criteria in Section 5.4. M1 should contribute across disciplinary contents; relevant M2 should add domain-specific predictive information beyond M1 and domain knowledge. These are between-person predictions from separately assessed capacities, not rescoring of the argumentation, source selection, or checking used to infer them. Domain-general and domain-specific contributions may overlap; neither exclusive effects nor an M total score is assumed. Evidence for these predictions must be distinguished from evidence for the M1/M2 structure and for the interactions in P4/P5 (Section 8.3)*.

**Proposition 4 (*S × M as a Between-Person Resource Interaction*).**  *Under comparable task and support conditions, the positive association of a pre-specified task-relevant M1 or M2 capacity with independently assessed judgment quality should be stronger at higher S. M provides the capacity to evaluate claims; S may support its goal-directed exercise. This proposed explanation is not itself established by the interaction. In the dashboard case, for example, differences in assessment-related M2 should be more strongly associated with defensible judgments about the learner among preservice teachers with higher S. Success is scored against the information and diagnostic standards specified in the reference dossier, not against the frequency of checking. This compares M–outcome relations at different levels of S, not only high–high with low–low profiles*.

**Proposition 5 (*A × M as a Between-Person Resource Interaction Under Ambiguity*).**  *In educational tasks with competing, unresolved interpretations, the positive association of a pre-specified task-relevant M1 or M2 capacity with independently assessed judgment quality should be stronger at higher A, under comparable task and support conditions. P4 concerns the conditions under which methodological capacity is exercised; P5 concerns its relation to judgment when competing interpretations remain unresolved. Both are between-person interaction hypotheses and require separate tests. A may limit closure driven by discomfort, allowing methodological capacity to inform the decision; the interaction would not establish this mechanism. In the dashboard case, assessment-related M2 should be more strongly associated with defensible judgments about the two explanations at higher A. The reference dossier determines whether a conclusion or qualification is warranted; prolonged openness earns no credit by itself. This prediction compares M–outcome relations across A levels, without implying a necessary A threshold or an advantage from A alone*.

**Proposition 6 (*Incremental Prediction Against Shared and Neighboring Accounts*).**  *For the independent judgment criteria in Section 5.4, A and pre-specified M components should add predictive information beyond a common S/SRL basis and relevant task knowledge. Separately, the S × M and A × M hypotheses require gain over the additive SAM model. SAM-based additions should also improve prediction beyond adequately represented neighboring accounts, including critical thinking in AI use (Lau et al., 2026). Each comparison tests a distinct claim. An informative absence of gain weakens that claim within the tested tasks and populations; conceptual integration alone does not establish predictive superiority*.

**Proposition 7 (*Conditional Validity of Digital Behavioral Indicators*).**  *For a pre-specified resource interpretation, theory-linked traces evaluated with task, support, and relevant affective information should show stronger convergence with separately elicited resource evidence not used to fit the mapping, and better discrimination from competing explanations, than a frequency-only account. These expectations should be pre-specified, and models should be compared under the same evaluation conditions on held-out participants or tasks, accounting for model flexibility. An adequately precise absence of the expected gain weakens that particular contextualized mapping claim, not all uses of traces. Predictive accuracy alone does not establish a resource interpretation; score comparability, fairness, and consequences require additional evidence (Section 5.1, Section 5.2, Section 5.3, Section 5.4 and Section 5.5)*.

**Proposition 8 (*Regulatory Locus and Development of Independent Regulation*).**  *Across a specified learning period, AI-supported arrangements that elicit learners’ own goal setting, monitoring, and strategy decisions are expected to yield larger gains in independently assessed regulatory capacity than otherwise comparable arrangements that routinely supply those decisions. The comparison concerns change in human capacity after the target regulatory support is withdrawn, not better products while support is available. Opportunities to practice and resume regulation are the proposed explanation, not proof supplied by a condition label. Persistent substitution is hypothesized to limit acquisition or maintenance; actual loss of an established capacity is a distinct, stronger claim. These are developmental predictions of SAM, not established general effects of AI*.

### Empirical Tests of the Model

The propositions (Table 3) require claim-matched designs rather than one all-purpose study. Each test should pre-specify the target resource or arrangement, task conditions, an independently scored criterion, analysis level, and time horizon. Person-level resources should be assessed separately from the target outcome; task manipulations do not randomize them. System affordances, information availability, and support conditions must be documented because they can constrain behavior. Where relevant, plausible simpler or nonlinear alternatives should be compared, accounting for model flexibility. For P4–P6, theoretically meaningful ranges for interaction effects or predictive gains should be pre-specified, and sufficient precision under the justified measurement model should be planned, accounting for measurement error; simulations can support this planning. A nonsignificant result with uncertainty spanning meaningful effects does not establish informative absence.

Resource distinctiveness must also be tested within SAM and against plausible neighbors such as conscientiousness or technology acceptance for S, anxiety or disengagement for A, and general ability, domain knowledge, or declarative data literacy for M. These are comparison questions, not an automatic covariate battery; process variables are not default controls. Shared task or method effects that fully account for apparent distinctions weaken the discriminant claim. No method is privileged by design: self-report and performance evidence are judged by their inferential role. Measurement invariance across relevant domains, educational levels, cultures, human–human versus human–AI configurations, and system versions requires separate justification. A COR-based resource-investment extension (Hobfoll, 1989; Hobfoll et al., 2018) should distinguish available resources, perceived scarcity, evaluative effort, and outcome; it is not P4 and does not imply an untested three-way interaction.

**Table 3:** Empirical Tests and Disconfirmation Conditions for SAM Propositions.

Proposition	Primary Test, Criterion, Comparison, and Interpretation
P1	Pre-assessed S should predict goal-appropriate delegation under specified task conditions. Score allocations against independently defined requirements, allow multiple defensible choices, and treat restricted opportunities to refuse or revise assistance as contextual constraints. A precise absence of the S–criterion relation weakens P1. Affective moderation is a separate extension and does not establish a causal S effect.
P2	Vary whether conflicting evidence leaves the judgment unresolved and test whether unwarranted closure increases more strongly at lower pre-assessed A. Document difficulty, information availability, and checking opportunities. Failure of the A × conflict relation challenges P2; deferral alone is not success.
P3	Assess M1/M2 separately from the target task and predict defensible judgment scored by domain-appropriate standards. M1 should generalize across disciplinary contents; relevant M2 should add within-domain information beyond M1 and domain knowledge. Failure of either contribution weakens the corresponding P3 claim, not all functions of M.
P4	Compare an additive resource model with the same model including S × M under comparable task and support conditions. The primary claim is between-person: the M–criterion relation should be stronger at higher S. A precise null interaction weakens P4. Within-person enacted regulation and cross-level extensions are separate questions.
P5	Under specified ambiguity, compare an additive model with A × M using the same independent criterion. The M–criterion relation should be stronger at higher A. Keep this test distinct from P2 and from any A × M × conflict hypothesis. In group tasks, individual judgments must be attributable separately from co-construction.
P6	Pre-specify whether the comparison concerns a common S/SRL-and-knowledge reference, additive SAM with interactions, or a neighboring account with and without SAM-based additions. Use the same independent criterion and comparable task conditions. For CT-in-AI-Use, justify total-score versus facet-level representation. Evaluate predictive gains on held-out or independent data with comparable measurement quality and model flexibility; lack of useful gain weakens only the tested incremental claim.
P7	For a pre-specified trace-to-resource interpretation, compare a contextualized mapping with a frequency-only account on held-out participants or tasks. Support requires stronger convergence with separately elicited resource evidence and better discrimination from competing explanations. Predictive accuracy alone does not establish construct validity, fairness, comparability, or appropriate use.
P8	Compare pre-specified regulatory arrangements across repeated sessions with baseline, immediate, and delayed independent performance after target support is withdrawn or faded. Less improvement is not itself loss of an existing skill; deskilling requires credible decline from an established baseline relative to an appropriate comparison trajectory. Randomization identifies arrangement effects, not the proposed practice mechanism; longitudinal scoring and attrition require examination. A precise absence of the predicted developmental advantage weakens P8.

## 7. SAM for Assessment

### 7.1. From System Outputs to Educational Decisions

Human-in-the-loop has two levels in the SAM framework. At the first level, learners or professionals regulate their own interaction with generative AI. At the second level, teachers, diagnosticians, advisors, and other educational professionals interpret AI-based assessments or learning analytics indicators. Formal human involvement is insufficient at both levels. The relevant question is whether goal setting, evaluation of uncertainty, selection of criteria of warrant, the possibility of challenge, and accountable final judgment actually remain with the human.

An AI-derived score is a machine-generated epistemic claim requiring interpretation. SAM hypothesizes that S, A, and M support its professional evaluation: S concerns the capacity to regulate attention, emotion, and action under time or automation pressure; A concerns willingness to tolerate unresolved diagnostic ambiguity; M concerns the capacity to select and apply methods to evaluate measurement models, data quality, misclassification, reference standards, bias, and validity limits. Possessing resources does not guarantee their exercise; the control-value perspective explains emotional responses to perceived control and value. Applying domain-specific criteria of warrant draws on M2, while assessment literacy also includes knowledge and other facets.

Three illustrations show the transition from assessment of SAM to SAM for assessment. In the dashboard case, the preservice teacher uses S to organize review rather than let the indicator or AI recommendation determine action. M1 compares low comprehension with incomplete activity records; M2 evaluates whether the records support the construct inference. A concerns willingness to retain unresolved explanations. The consequences of acting on uncertain evidence determine whether further review is warranted. Work samples and recording coverage inform the decision; success is a judgment or recommendation defensible against an independently prepared reference dossier.

For automated scoring or feedback, M2 concerns the construct, scoring rubric, reference material, bias, and intended use; M1 compares explanations for an anomalous result. Residual ambiguity may require qualification. Agreement with human ratings alone does not validate an intended score interpretation or use (American Educational Research Association et al., 2014). Success is a scoring or feedback judgment defensible against independently specified task standards.

In adaptive assessment, M2 concerns uncertainty around the ability estimate and classification boundary; S regulates whether further evidence is needed, while A concerns willingness to leave classification unresolved. A justified classification is assessed against independently specified decision standards using relevant supplementary evidence. A valid estimate does not demonstrate that subsequent support improves learning; that claim requires a separate intervention comparison with independent learning measures. Adaptivity does not itself imply generative AI.

Professional responsibility requires the capacity to judge an output’s scope, uncertainty, and legitimate use, with challenge or escalation when needed, rather than manually reproducing every calculation. A well-validated indicator may support routine low-risk decisions; high stakes, conflicting evidence, or visible model limitations warrant a lower threshold for human review. This is risk-sensitive calibrated reliance, not generalized skepticism. SAM leaves the threshold open: a future decision rule must consider consequences, reversibility, uncertainty in the evidence and validity argument, and the epistemic centrality of the delegated operation. Such operationalization of ultimate normative responsibility lies outside the present core architecture.

### 7.2. A Process-Oriented Assessment Culture Rather than an Illusion of Control

With generative AI, evaluation of the final output alone becomes less diagnostically informative. In essays, presentations, or oral reports, the product increasingly provides an ambiguous basis for inferring which cognitive and judgmental processes were actually performed. Assessment should therefore combine process traces, brief oral follow-ups, source logs, reflective statements, and transfer or variation tasks more systematically. The aim is not comprehensive surveillance but triangulation: have learners understood the concepts, decisions, and evaluation criteria, and can they transfer them to changed situations (Horz & Czora, 2026)?

Economical process assessment can use a short disciplinary conversation, three-sentence reflection (“This is what I thought myself—this is what the AI added—this is what I checked”), targeted peer feedback, or a transfer question; these may be more informative than extensive full-process logs.

Agents, multimodal, and potentially wearable systems are expected to make assistance harder to detect, intensifying the illusion of control. Durable solutions likely depend on instructional design rather than surveillance: make thinking visible, require independent judgment, and cultivate reflective AI use rather than an invisible parallel practice.

Process traces should make independent goal setting, evaluation, and judgment assessable, not exhaustively reconstruct disclosure of AI assistance. Assessment culture is therefore a validity issue: assess the performance about which the intended inference is made.

### 7.3. Educator AI Readiness

Educator readiness includes cognition, ability, vision, and ethics (Wang et al., 2023). SAM hypothesizes that educators’ S, A, and M support warranted interpretation of AI evidence. Professionals also need knowledge to diagnose and foster learners’ resources: SRL supports this dual role for S (Dignath, 2021; Karlen & Dignath, 2026; Karlen et al., 2020), while A and M remain hypotheses. Operationalization must fit sectors: developmental diagnosis and assessment in schools; scholarly independence, methodology, research ethics, and publication in higher education; and workplace decisions under time pressure in continuing education.

Professional development should combine cases involving conflicting indicators, bias, model limits, and appropriate reliance with reflection on lost control, mistrust, relief, and instructional design. SDT suggests meaningful professional discretion, evaluative competence beyond compliance, and collegial calibration (Ryan & Deci, 2020). Success should be evaluated through independent cases, authentic teaching/diagnostic transfer, and longitudinal change, rather then acceptance, self-efficacy, checking frequency, or supported product quality alone.

Professionals need not master every new system; their expertise specifies learning/diagnostic goals, detects substitution of target competences, and judges evidential warrant. Domain and diagnostic knowledge supply alternative indicator explanations. Higher education and research can apply institution-specific authorship, integrity, and accountability standards rather than tool-specific training (Zyphur, 2026).

### 7.4. Organizational Conditions for AI Readiness and Valid Use

Institutional conditions determine whether SAM resources can be exercised and whether observed behavior can be interpreted validly. Policies should specify permitted and documented AI uses, accountable roles, the evidence that users may inspect, and routes for challenge or escalation, rather than rely on ad hoc technical guidance alone (Kaşarcı et al., 2025). Time, access, and professional support also shape whether checking is feasible. These conditions are contextual affordances and constraints, not additional SAM resources. In higher education, Kaşarcı et al. (2025) synthesize recurring gaps in coordination, ethics literacy, and policy evaluation, while also noting that much of the evidence is based on self-report or normative policy analysis. The review therefore supports treating governance as an implementation and validity context, not as proof that a particular governance structure improves S, A, or M.

Technical infrastructure belongs to the same validity context. Systems used for learning or diagnosis should make relevant evidence inspectable, uncertainty and model version identifiable where feasible, and consequential decisions contestable or reviewable. Research-oriented examples include verifiable citations, reproducibility at known model versions, and auditability (Zyphur, 2026). These features do not guarantee good judgment, but their absence can restrict the exercise and observation of S or M. Validation studies should therefore document access rights, logging and documentation rules, update cycles, and the actual opportunities users have to inspect, revise, or escalate a result.

Governance combines coordination and local adaptability: institution-wide safeguards cover consequential use, responsibility, documentation, review, and equity; disciplines specify evidence and assistance standards. Kaşarcı et al. (2025) recognize centralized/distributed approaches and reasonable provisional or disciplinary rules under uncertainty; incoherent roles, standards, or accountability create risk. DOIT and Instats support cyclical development across didactics, organization, individuals, and technology (Horz & Ulrich, 2013; Horz et al., 2002; Zyphur, 2026). Revise policies using misclassification, appeals, subgroup disparities, user experience, and updates. This remains a design proposal with limited comparative-effectiveness evidence.

## 8. Educational Implications and Research Agenda

### 8.1. Curricular Design Principles

The educational implications should be treated as testable design assumptions implemented under explicit institutional conditions. Intervention designs can make delegation decisions visible, create encounters with conflicting or incomplete outputs, and require domain-defined evaluation practices. Encounters with discrepant evidence can serve conceptual change when alternatives become intelligible, plausible, and fruitful (Posner et al., 1982). In practice, however, the same task may support or suppress actions related to S, A, or M depending on available time, access to evidence, permitted tools, and routes for help or escalation. Studies and curricula should therefore specify these conditions rather than attribute constrained behavior solely to the learner. For learning analytics, process indicators remain interpretable only relative to the task phase, intervention, target competence, and the institutional opportunities to inspect and contest the system.

### 8.2. Developmentally Appropriate Specification

Developmental appropriateness is both an intervention and a validity issue. In primary education, teachers can first model how learners generate their own solution before using AI and then compare it with a system suggestion. In secondary education, learners can take increasing responsibility for generating their own hypotheses, triangulating sources, and completing transfer tasks. In higher education and research, independent question formulation, methodological validation, authorship, and research-integrity issues move to the foreground; here, Zyphur’s (2026) distinction between “labour” and “judgement” provides a relevant task-specific specification. In professional contexts, the same principles must be tested under time pressure, real stakes, and organizational responsibility. These examples mark research designs, not fixed stage standards.

Developmental comparisons require more than mean comparisons: high levels of external support appropriate in primary education may indicate insufficient independence in higher education. Invariance needs theoretical justification because identical behavior may represent different constructs across levels. The scaffolding density, task complexity, and expected autonomy must be specified.

### 8.3. Domain-General and Domain-Specific Methodological Competence as a Test of M

The M1/M2 distinction remains a structural and predictive hypothesis, not merely a labeling choice. Testing it requires M1 assessment across disciplinary contents and M2 assessment within the relevant domain, followed by separate target tasks scored against domain-appropriate standards. M1 should show interpretable cross-domain prediction that is not fully accounted for by general verbal or cognitive ability; M2 should add within-domain information beyond M1 and independently assessed domain knowledge. Task, method, partner, or support effects that fully explain apparent components would weaken the structural claim, while a differentiated structure alone would not establish P3. Human–human and human–AI comparisons additionally require comparable score interpretations and individual attribution. Cross-domain prediction should not be called training transfer without an appropriate intervention and comparison.

### 8.4. Educational Equity and Epistemic Stratification

Within SAM, educational equity is closely linked to construct validity. One empirically testable causal chain is that unequal access to high-performing systems, unequal opportunities to learn S, A, and M, and unequal domain-specific support may produce diverging trajectories in the quality of AI use and, over time, in learning, expertise, and judgment. Observable differences in use or performance should not be prematurely reified as stable person characteristics; access, learning opportunities, and institutional factors must be considered analytically.

An accelerated Matthew effect remains a risk hypothesis, not an already established general consequence of generative AI. It builds on well-known findings concerning cumulative advantage in education and competence development (Merton, 1968; Stanovich, 1986; Walberg & Tsai, 1983) but requires independent testing for generative AI.

Analytics traces differ with systems and opportunities; apparent S or M differences may be contextual. It is crucial to test whether comparable indicator values warrant comparable interpretations across access conditions and whether support decisions reduce or amplify disparities.

Equity-oriented implementation therefore requires more than nominally equal access. Institutions should examine whether learners have comparable opportunities to obtain appropriate systems, disciplinary support, independent practice, explanation, and appeal. Assessment rules should prevent unequal access to more capable tools from becoming an unexamined source of performance differences, and high-stakes AI-derived indicators should be monitored for group- and context-sensitive misclassification. Where disparities arise, the institutional response should distinguish measurement bias, access differences, and unequal learning opportunities before treating the pattern as an individual deficit.

### 8.5. Priorities for Future Research

Future research should first test the resource distinctions, interaction claims, and neighboring-model comparisons specified in Section 5 and Section 6. Contextual accounts from CVT, SDT, and COR require separate tests of appraisals, emotions, motivation, resource availability, and enactment rather than combining them in a single comprehensive model. For M1, reflective, formative, and multidimensional representations remain alternatives whose indicator relations must be justified before model fit is compared. Event-proximal observations can address process questions without substituting for between-person resource tests.

The agenda must also connect measurement, development, and institutions. Changes in system affordances or model versions can mimic changes in S, so P7 validation should be linked to P8 follow-ups and to documented human–human versus human–AI configurations. Institutional studies should measure time for checking, access to evidence, responsibility, review routes, and system documentation rather than assume every policy variable is a moderator. Professional-development and governance interventions should be evaluated against independent decision quality, transfer, fairness, and implementation outcomes. SAM’s scope and its enabling conditions must be established empirically rather than inferred from policy adoption.

## 9. Overall Conclusion: SAM as a Testable Theory of Epistemic Control

SAM is a bounded model of three person-level resources—S, A, and M, with hypothetical M1/M2 differentiation—for preserving human judgment in AI-supported learning, assessment, and professional decisions. It specifies which person-level resources condition warranted evaluation of AI-generated claims and how they interact; domain knowledge, ethics, social interaction, system affordances, and governance belong to broader readiness and context rather than to this core. Epistemic control remains an interaction-level property; preserving it and safeguarding intellectual sovereignty are normative aims, not SAM scores.

The model is falsifiable. It would be weakened if the resources were not distinguishable or incrementally informative; if the specified S × M and A × M relations failed in adequately precise tests; if M1/M2 reduced to simpler alternatives; if contextualized trace mappings added no defensible resource evidence; or if the predicted P8 developmental differences did not emerge. Supported product quality, AI-use frequency, or policy adoption alone do not establish these claims. Affective, motivational, resource-investment, cultural, and institutional conditions require separate tests.

Inferences from assessment and learning analytics data require validated construct representation, alternative explanations, comparability, fairness, and intended use. Human-in-the-loop requires inspection, challenge, revision, and contestability, with success scored by independent task/domain standards. SAM’s contribution depends on whether it improves explanation and prediction beyond shared and neighboring accounts, including critical thinking in AI use, while limiting the model to three person-level resources.

## Figures and Tables

**Table 1:** SAM Resources, Candidate Observations, and Validity Threats.

SAM Component	Target Resource	Candidate Observations in AI Tasks	Required Contextual Information	Central Validity Threat
S	Capacity to regulate goals, attention, emotions, strategies, and delegation	Planning before AI use; goal-related revisions; switching between sources; changes in delegation or non-use, with reasons	Task goals, prior knowledge, difficulty, learning or application phase, support, and system affordances	Frequent activity may reflect trial and error, task difficulty, or compliance rather than regulatory capacity
A	Disposition-like willingness to tolerate unresolved epistemic ambiguity	Responses to conflicting interpretations; reported willingness and reasons for closure; exploration, uncertainty notes, and decision latency	Perceived ambiguity, emotions, stakes, available information and options, domain knowledge, and task demands	Delay may reflect confusion, avoidance, or missing knowledge; discomfort does not by itself establish low A
M1	Capacity to compare, challenge, and integrate epistemic contributions across domains, including in collaborative work	Source and perspective comparisons; reasons, counterarguments, uptake of others’ contributions, and common-ground or integration moves	Task role, prior knowledge, discourse structure, partner contributions, group composition, and support	Fluent argument or agreement may reflect verbal skill, conformity, or partner support rather than individual M1
M2	Capacity to select and apply domain-specific procedures and criteria for evaluating claims	Source corroboration, justified triangulation, counterexamples, boundary-case tests, and appraisal of data provenance or inference limits	Domain knowledge, source and test quality, evaluation standards, access to evidence, and available support	Checking may follow a script without methodological understanding; correct outcomes may reflect prior knowledge or assistance

Note. S = self-regulation; A = ambiguity tolerance; M1 = domain-general epistemic-methodological competence; M2 = domain-specific methodological competence. ML = machine learning. Entries are candidate observations, not validated scores or exclusive markers. Frequency is not quality; resource assessment and independent outcome assessment serve different purposes. ML-based classroom measurement illustrates the construct-coverage and contextual issues involved, not validation of SAM indicators (Lazarides et al., 2026).

**Table 2:** Multimethod Assessment of SAM Resources.

Resource	Candidate Assessment	Task-Linked Evidence	Inference to Evaluate
Self-regulation (S)	Selected SRL self-report scales (e.g., MSLQ); planning and adaptation tasks	Experience sampling, think-aloud, SJTs and traces linked to goals, delegation demands and support; separate control/value appraisals and emotions	Distinguish reported strategies, episode-level regulation and capacity; examine goal-relevant adaptation across tasks against explanations based on difficulty, prior knowledge or compliance
Ambiguity tolerance (A)	MSTAT-II or another justified dispositional measure; epistemic-ambiguity vignettes	Contrastive interpretations; perceived ambiguity, willingness, emotions and reasons for closure recorded separately	Test epistemic fit and alternatives such as confusion or avoidance; neither latency nor justified deferral alone measures A
Methodological competence (M1): domain-general epistemic-methodological competence	Argumentation and collaboration tasks; epistemic-strategy knowledge as supplementary evidence	Matched requirements across domains and partners: perspective integration, counterargument, common ground and AI-supported co-construction	Attribute contributions to the person; compare integration quality across domains while considering verbal skill, partner and support effects
Methodological competence (M2): domain-specific methodological competence	Methods-knowledge tests and domain-case performance tasks	Erroneous or incomplete AI outputs evaluated with explicit domain-specific criteria	Infer application capacity from warranted choices and explanations, not counts; examine domain knowledge, prompting and task support

Note. S = self-regulation; A = ambiguity tolerance; M1 = domain-general epistemic-methodological competence; M2 = domain-specific methodological competence. SRL = self-regulated learning; MSLQ = Motivated Strategies for Learning Questionnaire; MSTAT-II = Multiple Stimulus Types Ambiguity Tolerance Scale-II; SJT = situational judgment test. Examples are candidates, not a fixed battery. Appraisals and emotions describe potential conditions or processes, not additional SAM resources. M1/M2 labels do not establish separable scores; evidence across tasks and methods must test that claim.

## Data Availability

No new data were created or analyzed in this theoretical article.
